# Digesting the crisis: autophagy and coronaviruses

**DOI:** 10.15698/mic2020.05.715

**Published:** 2020-05-04

**Authors:** Didac Carmona-Gutierrez, Maria A. Bauer, Andreas Zimmermann, Katharina Kainz, Sebastian J. Hofer, Guido Kroemer, Frank Madeo

**Affiliations:** 1 Institute for Molecular Biosciences, NAWI Graz, University of Graz, Graz, Austria.; 2 BioHealth Graz, Graz, Austria.; 3 Centre de Recherche des Cordeliers, Equipe labellisée par la Ligue contre le cancer, Université de Paris, Sorbonne Université, INSERM U1138, Institut Universitaire de France, Paris, France.; 4 Metabolomics and Cell Biology Platforms, Institut Gustave Roussy, Villejuif, France.; 5 Pôle de Biologie, Hôpital Européen Georges Pompidou, AP-HP, Paris, France.; 6 Suzhou Institute for Systems Medicine, Chinese Academy of Medical Sciences, Suzhou, China.; 7 Karolinska Institute, Department of Women's and Children's Health, Karolinska University Hospital, Stockholm, Sweden.; 8 BioTechMed Graz, Graz, Austria.

**Keywords:** COVID-19, SARS, SARS-CoV-2, MERS, coronavirus, virophagy, inflammation, immunity

## Abstract

Autophagy is a catabolic pathway with multifaceted roles in cellular homeostasis. This process is also involved in the antiviral response at multiple levels, including the direct elimination of intruding viruses (virophagy), the presentation of viral antigens, the fitness of immune cells, and the inhibition of excessive inflammatory reactions. In line with its central role in immunity, viruses have evolved mechanisms to interfere with or to evade the autophagic process, and in some cases, even to harness autophagy or constituents of the autophagic machinery for their replication. Given the devastating consequences of the current COVID-19 pandemic, the question arises whether manipulating autophagy might be an expedient approach to fight the novel coronavirus SARS-CoV-2. In this piece, we provide a short overview of the evidence linking autophagy to coronaviruses and discuss whether such links may provide actionable targets for therapeutic interventions.

## INTRODUCTION

The ongoing outbreak of the respiratory disease COVID-19 (Coronavirus Disease 2019) is endangering individuals, governments and societies around the world. Indeed, this pandemic has unprecedented global consequences at the economic and social levels as it challenges the medical and research communities. The first case of COVID-19 was reported in December 2019 in Wuhan (China) [1] and, despite increasingly drastic efforts to contain the disease, the infection rapidly spread across the planet, leading the WHO to declare it, first, a global health emergency (in January 2020) and eventually a pandemic (on March 11, 2020) [2, 3]. As of April 30^th^ 2020, more than 200 countries and territories have reported COVID-19 cases with a total of over three million confirmed cases and more than 220.000 confirmed deaths [4]. In the current absence of effective treatments and vaccines, these numbers will continue to grow inexorably. The main clinical features of COVID-19 (fever, dry cough, pneumonia) manifest after an average incubation period of approximately 5 days [5, 6] with severe cases advancing towards severe acute respiratory syndrome (SARS) [7–9]. COVID-19 was quickly shown to be caused by a novel coronavirus (CoV) that was named SARS-CoV-2 [7, 10].

SARS-CoV-2 is structurally related to the members of the *Coronaviridae* family in the order *Nidovirales* [10, 11]. CoVs are enveloped viruses with a single-strand, positive-sense RNA genome and are separated into four genera according to their genome characteristics: α-CoV, β-CoV, γ-CoV, and δ-CoV [12, 13]. Until the emergence of COVID-19, six human pathogenic CoVs (hCoVs) had been identified: four non-SARS-like hCoVs (HCoV 229E, NL63, OC43, and HKU1), which do not cause major (fatal) epidemics, and two SARS-like hCoVs (SARS-CoV and Middle East respiratory syndrome coronavirus MERS-CoV) [14]. Both SARS-like hCoVs, which belong to the β-CoV genus, have already caused major outbreaks and health emergencies in China (SARS-CoV, 2002-2003) as well as in the Middle East (MERS-CoV, 2012) [15]. The novel SARS-CoV-2 increases the number of known hCoVs to seven, all of which have a zoonotic origin, meaning that they originate from vertebrate animals (possibly bats for SARS-CoV-2) and have reached humans via interspecies transmission [16].

## AUTOPHAGY AS A PROTECTIVE CATABOLIC PROCESS

Given the devastating scenario that COVID-19 has already generated, a multitude of potential approaches against SARS-CoV-2 are currently being conceived. In that sense, we think that the manipulation of autophagy might be worth exploring. Macroautophagy (hereafter referring to autophagy) is an intracellular catabolic mechanism that regulates the elimination of unwanted, superfluous or defective cell components [17]. In this process, the targeted molecules/organelles are enclosed in double-membrane vesicles called autophagosomes, which eventually fuse with lysosomes for degradation of the delivered contents. This process is induced upon energy deprivation and/or physical activity in order to secure sufficient nutrient supply and is thus often referred to as a cellular self-digestion system. The compounds resulting from lysosomal degradation (e.g., fatty acids, amino acids) may be released into the cytoplasm for the generation of new macromolecules and/or bioenergetics reactions. Autophagy, therefore, represents a recycling mechanism under circumstances of low resource availability [17].

The digestion of damaged and thus potentially detrimental cargo also increases the fitness at the cellular and consequently at the organismal level. Accordingly, the induction of autophagy by dietary interventions (e.g., caloric restriction, fasting regimens), behavioral cues (e.g., exercise) or pharmacological agents (e.g., caloric restriction mimetics like spermidine, rapamycin, resveratrol or specific chalcones [18–22]) has been consistently linked to beneficial effects on health [23]. In turn, reduced autophagic capacity is connected to aging progression and a number of pathologies including cardiovascular disease, cancer and neurogeneration [24].

The autophagic machinery can retrieve its cargo in bulk, but it can also selectively target distinct intracellular components and organelles via specific adapter proteins that interact with both the individual substrates and the autophagosomes [25]. Specifically, these autophagy adaptors (e.g., p62/SQSTM1, optineurin) contain both a ubiquitin-binding domain (UBD) and an LC3-interacting region (LIR). The UBD recognizes ubiquitin tags decorating the specific target, while the LIR allows binding of LC3 proteins attached to nascent phagophores, the structures that eventually close to form autophagosomes [26]. Accordingly, autophagic subforms have been defined that describe, for example, the specific removal of damaged mitochondria (mitophagy), the disposal of protein aggregates (aggrephagy), the digestion of lipid droplets (lipophagy), etc [27].

Yet another selective autophagy subroutine is xenophagy, the specialized elimination of intracellular pathogens, including fungi, bacteria and viruses [28]. The xenophagic disposal of viruses (sometimes also termed virophagy) has been described for different viral pathogens, including human immunodeficiency virus-1 (HIV-1) [29, 30] and herpes simplex virus-1 (HSV-1) [31, 32]. However, many viruses (including HIV-1 and HSV-1) and other pathogens have also evolved strategies to escape or inhibit autophagy and sometimes even to manipulate the autophagic machinery for their replicative benefit [33]. Thus, autophagy emerges as a crucial element in the evolutionary-driven arms race of host against pathogen. This further underlines the significance of xenophagy as a central antimicrobial mechanism, but also limits the potential of autophagy induction to fight infections.

## CORONAVIRUSES AND AUTOPHAGY

The question whether autophagy induction might be beneficial to specifically counteract SARS-CoV-2 infection cannot be answered at this point. However, the existing data on other CoVs suggest that – despite some conflicting results – autophagy induction might be a valid approach that should be subjected to further evaluation. The autophagy-lysosomal system does indeed seem to play a central role during the infection with different CoVs, including SARS-CoVs [34–37]. Nevertheless, the diverse putative roles of autophagy during viral infection [38] require that two opposite aspects be covered by this overview: (i) Is there evidence for autophagy being used for the survival or replication of CoVs (proviral effects)? (ii) Can autophagy induction reduce cellular infection, and does the virus actively inhibit autophagy (suggesting antiviral effects for autophagy)? Of note, cellular manipulation of autophagic levels during infection may also reflect desperate attempts of the cell to reestablish homeostasis, either through restriction of viral entry by actively shunting endocytosis/endosomal trafficking (possibly resulting in autophagy reduction as a side-effect) [39] or to counteract virally induced cell death by increasing cytoprotective autophagy. As a third major aspect, (iii) the autophagic effects on antiviral immunity must be taken into consideration. Over the last 15 years, a number of studies have addressed these issues in the context of the “old” CoVs, those that historically precede SARS-CoV-2.

### Can autophagy be highjacked for CoV replication?

CoVs rely on the formation of replication complexes at double membrane vesicles (DMVs), where viral genome replication and transcription occurs [40, 41]. As it may be at least partly the case for autophagosomes, DMVs probably depend on ER-derived membranes for their generation [40, 42]. Therefore, it was initially suspected that CoVs might usurp the autophagosomal machinery for DMV formation. In fact, an early study using *ATG5* knockout cells implied that autophagy may promote mouse hepatitis virus (MHV) replication via formation of DMVs [43]. In a follow-up work, the same authors reported the co-localization of specific replication proteins (nsp2, nsp3, nsp8) with endogenous LC3, suggesting a connection between replication and autophagosome formation [44]. Other studies reported that the porcine CoVs transmissible gastroenteritis virus (TGEV) [45] and porcine epidemic diarrhea virus (PEDV) [46] may induce autophagy to promote replication. However, the link between autophagy and replication of CoVs has been challenged by a number of different reports, showing that deletion of the essential autophagy genes *ATG5* or *ATG7* does not affect the replication of MHV [47, 48] or SARS-CoV [49]. Furthermore, the co-localization of LC3 (or GFP-LC3) with the SARS-CoV RNA replication complex could not be confirmed [50]. A study using pharmacological induction (rapamycin) or genetic inhibition (knockdown of LC3, ATG5, or ATG7) of autophagy even demonstrated that TGEV replication is negatively regulated by autophagy [51]. In addition, rapamycin reduced infectivity of PEDV [52]. Altogether, although some conflicting results exist, autophagy may not be primarily engaged in promoting CoV replication.

**Figure 1:**
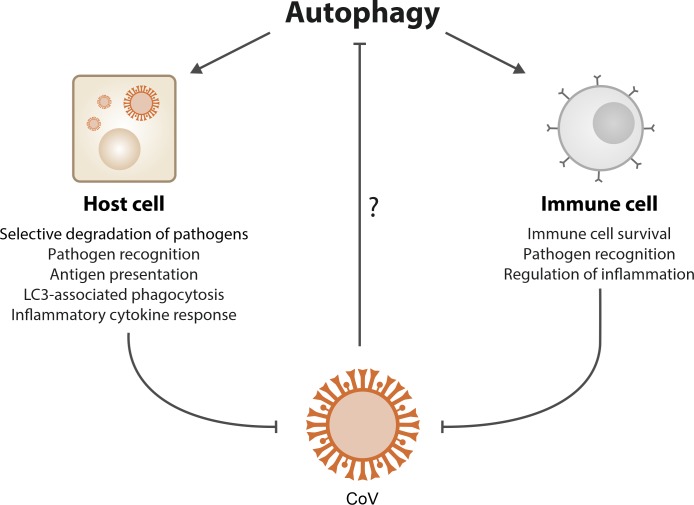
FIGURE 1: Possible protective mechanisms of autophagy against coronaviruses. Autophagy may counteract viral infection by executing and supporting different antiviral pathways in infected host cells as well as by promoting the function and survival of immune cells. Therefore, an increase in autophagic flux may be effective against coronaviruses (CoVs). Accordingly, CoVs may have developed strategies to limit cellular and organismal immunity by preventing autophagy.

However, single components of the autophagic machinery may be hijacked independently of their activity in autophagic processing. Non-lipidated LC3, for instance, is used for membrane derivation for DMVs in CoVs, and downregulation of LC3 but not inactivation of autophagy, counteracts CoV infection [47]. Notably, some components of the canonical autophagy machinery can target invading pathogens by promoting phagosome-to-lysosome fusion [53]. During LC3-associated phagocytosis (LAP), for instance, which does not involve sequestration in autophagosomes, LC3 is recruited to phagosome membranes to facilitate lysosomal fusion and degradation [54]. In addition, it has been proposed that non-canonical autophagy and intruding viruses, both of which can be inhibited by the fungal compound brefeldin A, may converge in the retrieval of membranes [55]. Non-canonical autophagy includes ATG5- and ATG7-independent as well as Beclin-1-independent autophagy [56, 57]. Altogether, these observations add further layers of possible modulation of autophagic processes in trying to attenuate viral infection **(Figure 1)**.

### Does autophagy counteract CoV infection?

Intriguingly, a body of evidence indicates that CoVs may actually inhibit autophagy, which in turn teleologically suggests an antiviral role for autophagy. Accordingly, a number of studies have shown that increasing autophagic capacity may be beneficial against infection **(Figure 1)**. A temporal kinome analysis of MERS-CoV-infected hepatocytes demonstrated selective activation of the ERK/MAPK and PI3K/AKT/mTOR signaling responses, both of which have inhibitory effects on autophagy [58]. Accordingly, pharmacological inhibition of these pathways was able to inhibit MERS-CoV infection [58]. Moreover, although one of the central replicase proteins (nsp6) of avian infectious bronchitis virus (IBV) seems to promote autophagosome formation at the omegasome level [59], nsp6 also limits further autophagosomal expansion [60], thus compromising autophagic delivery of viral components to lysosomes. Interestingly, nsp6 is also present in other CoVs, including SARS-CoV-2 [61].

Overexpression of the protease PLP2 of SARS-CoV and MERS-CoV in different cell lines inhibited autophagosome-lysosome fusion and autophagic flux [62]. A recent report corroborated the autophagy-inhibitory activity of MERS-CoV and showed that induction of autophagy can reduce MERS-CoV replication [37]. Specifically, the authors found that pharmacological inhibition of the ubiquitin ligase SKP2 increases the levels of Beclin-1, a central regulator of phagophore formation [37]. SKP2 executes lysine-48-linked poly-ubiquitination of Beclin-1, targeting it for proteasomal degradation, the inhibition of which promoted autophagy and efficiently reduced MERS-CoV replication. This work also shows that ectopic expression of at least three viral proteins (nsp6, p4b, p5) restricts autophagy [37]. While nsp6 may limit autophagosome expansion (see above), p4b inhibits RNAse L activation (a pro-autophagic event [63]), and p5 has been connected to the blockage of IFN-β induction [63, 64], which itself may be linked to autophagy [65]. Accordingly, deletion of p4b or p5 resulted in reduced MERS-CoV growth, although disputing some previous observations, which the authors explain by methodological differences [37]. Thus, the group-specific accessory proteins, which by definition are not essential for viral replication but are involved in the modulation of host cells and immune evasion [66, 67], may represent targets for reducing the autophagy-inhibitory effects of CoVs.

The FDA-approved anti-malarial drugs chloroquine and hydroxychloroquine have been suggested to be repurposed for the treatment of COVID-19 [68–70], but this remains widely controversial [71–73]. Although chloroquine is a lysosomotropic agent that blocks autophagic degradation, possibly by impairing autophagosome fusion with lysosomes [74], the putative effects on autophagy may not be necessarily causal for the antiviral activity. In fact, endosomal acidification after endocytosis is critical for SARS-CoV-2 entry [75], and chloroquine inhibits this acidification [76]. In addition, chloroquine limits terminal glycosylation of the metallopeptidase ACE2, the functional receptor for SARS-CoV and SARS-CoV-2 cell entry [68, 75, 77]. Non-glycosylated ACE2 seems to interact less efficiently with the SARS-CoV spike protein, thus reducing viral entry [78]. These modes of action would target the virus upstream of autophagy, making it unlikely that autophagy modulation contributes to the outcome of chloroquine treatment at that point. Additionally, chloroquine has been shown to induce autophagy-independent effects, for instance, Golgi disorganization [74] and pulmonary vasodilation [79] that may contribute to its controversial clinical activity. Interestingly, the net effects on autophagy may differ depending on parameters like cell type and dose: for instance, chloroquine-mediated lysosomal stress may promote the nuclear translocation of the pro-autophagic transcription factors TFEB and TFE3 [80, 81]. Altogether, further clinical work must clarify if chloroquine and hydroxychloroquine have major effects on COVID-19 and at which (early?) phase of the disease these drugs should be administered. If chloroquine or its derivative turned out to be clinically useful against COVID-19, it will be important to understand to which extent such effects are connected to the modulation of autophagy.

Indeed, repurposing of known (especially currently FDA-approved) drugs might offer a welcome shortcut to rapidly developing treatments against COVID-19. Obviously, even in the present period of desperate search for pharmacological treatments, the efficacy (and lack of side-effects) of anti-COVID-19 treatments needs to be proven by clinical studies subjected to rigorous scientific scrutiny. While data regarding such attempts remain preliminary at this stage, several currently available preprints allow the speculation of autophagy as a possible druggable target. For instance, a recent preprint mapping the SARS-CoV-2 interactome in human host cells identifies several host factors connected to autophagy [82]. Amongst others, the authors find viral-human interactions with proteins directly modulated by mammalian target of rapamycin complex 1 (mTORC1) [82], a master regulator of cell proliferation and autophagy known to be affected by other viruses [83, 84].

Intriguingly, another recent preprint presents *in vitro* data showing that SARS-CoV-2 infection restricts autophagy and that, in turn, pro-autophagic compounds - including spermidine - may inhibit viral propagation [85]. Admittedly, spermidine has a plethora of physiological functions, including regulation of RNA-to-protein translation, as well as amelioration of RNA and DNA stability [86], and these effects need further investigation, specifically in relation with a possible autophagy induction. In fact, the role of spermidine during infection may be pleiotropic. On the one hand, spermidine is an autophagy inducer [18, 87–90] and may promote viral xenophagy; also, it may contribute to endosomal pH buffering [91] and thus block viral entry. On the other hand, polyamines are essential for viral replication [92], and the overexpression of spermidine/spermine-N(1)-acetyltransferase by the host, which drives spermidine (and spermine) depletion, is a common cellular response to viral infections, including that of RNA viruses [93]. This underlines once more that any pharmacological candidate for the fight against SARS-CoV-2 should be examined for its (possible) pleiotropy during infection.

### The role of autophagy on immune signaling

Autophagy directly impacts immune signaling, and autophagy induction hence may counteract CoVs by promoting immunological defense mechanisms **(Figure 1)**. For instance, autophagy regulates, and can be regulated by, so-called pattern recognition receptors (PRRs), which sense molecules specifically derived from microorganisms (pathogen-associated molecular patterns, PAMPs) [94, 95]. PRRs are present in various cells of the innate immune system and many epithelial cells [95]. There, they are distributed at diverse locations (either at the cell surface or in the cytoplasm), providing a recognition system for different life cycle phases of a given pathogen [95]. PAMP-induced signaling triggers a multitude of pathways, including pro-inflammatory responses, that result in the synthesis of immunostimulatory molecules including cytokines, chemokines and immunoreceptors [94].

Autophagy further assists immune responses by processing and supporting the presentation of antigens on major histocompatibility complex (MHC) class II, responsible for loading extracellular antigens [96, 97]. In addition, autophagy can deliver exogenous viral antigens into MHC-I molecules for cross-presentation (the MHC-I pathway is usually employed for loading endogenous antigens) [97, 98]. Moreover, autophagy directly impacts the activation, proliferation and differentiation of immune cells [99]. Amongst others, autophagy has been connected to the differentiation of CD8^+^ T-cells into cytotoxic T lymphocytes, to dendritic cell and B cell development, to plasma cell differentiation, and to macrophage differentiation, therefore covering multiple instances of the innate and adaptive immune systems [99]. Of note, autophagy activation by caloric restriction mimetics (including hydroxycitric acid and spermidine) also improves anticancer immunosurveillance [100]. In addition, the general cellular pro-survival effects of autophagy extend to several immune cell types. Pharmacological autophagy induction by spermidine [87], for instance, has been demonstrated to promote the maintenance and survival of memory CD8^+^ T cells [101] as well as to counteract B lymphocyte senescence [102].

The interplay of autophagy and inflammation is complex, since it does not only encompass positive but also negative regulatory mechanisms. For example, autophagy can digest the interleukin precursors produced by inflammasomes (e.g. pro-IL-1), but also directly target inflammasome components (e.g. NLRP3, AIM2 and ASC) [103]. In sum, autophagy ensures acute inflammatory responses while preventing excessive and prolonged hyperinflammation.

Finally, it should be noted that autophagy may have paracrine functions in the form of unconventional secretion [104], thus adding yet another layer of non-cell autonomous effects. In the context of viral infections, this mechanism may be used to induce protective responses in cells neighboring an infection site [105, 106]. In primary human placental trophoblasts, for instance, autophagy induction promotes packaging and exosome-dependent delivery of specific miRNAs, which induce autophagy in non-placental recipient cells, conferring resistance to a variety of viruses [105]. This may be (one of) the mechanism(s) through which placental and maternal cells optimize their defense against viral infections during pregnancy.

## CONCLUSIONS

The existing evidence allows the speculation that autophagy induction might counteract CoV infection at different levels, although more specific data are certainly required. As mentioned above, the restriction of calorie intake is the most robust method to induce autophagy. However, the fight against acute infections also requires sufficient energy supply, suggesting that autophagy induction via caloric restriction or fasting regimens may be counterproductive, at least in the short-term during (as well as shortly before) infection. Therefore, caloric restriction mimetics [107, 108], natural or synthetic compounds with the ability to induce autophagy, may circumvent this problem.

Irrespectively of whether autophagy modulation will eventually be part of the strategies against COVID-19, the current pandemic outbreak is a shocking reminder that emerging (and re-emerging) infectious pathogens are (and will be) a major challenge. In view of the exposed vulnerability of our medical structures and socioeconomic well-being, this pandemic underlines how essential it is to further establish and secure global healthcare as well as to promote and extend robust research against infectious diseases.

## Abbreviations:

CoV: – coronavirus;
COVID-19: – Coronavirus Disease 2019;
DMV: – double membrane vesicle;
hCoV: – human pathogenic CoV;
HIV: – human immunodeficiency virus;
HSV: – herpes simplex virus;
LIR: – LC3-interacting region;
MERS: – Middle East respiratory syndrome;
MHC: – major histocompatibility complex;
MHV: – mouse hepatitis virus;
PAMP: – pathogen-associated molecular pattern;
PEDV: – porcine epidemic diarrhea virus;
PRR: – pattern recognition receptor;
SARS: – severe acute respiratory syndrome;
TGEV: – transmissible gastroenteritis virus;
UBD: – ubiquitin-binding domain.
